# Effectiveness of a combined problem-based learning and flipped classroom teaching method in ophthalmic clinical skill training

**DOI:** 10.1186/s12909-022-03538-w

**Published:** 2022-06-23

**Authors:** Anan Wang, Ruihan Xiao, Chun Zhang, Lin Yuan, Nana Lin, Lu Yan, Yaohua Wang, Jinhai Yu, Qin Huang, Puying Gan, Chao Xiong, Qihua Xu, Hongfei Liao

**Affiliations:** Department of Ophthalmology, Affiliated Eye Hospital of Nanchang University, Jiangxi Clinical Research Center for Ophthalmic Disease, Jiangxi Research Institute of Ophthalmology and Visual Science, Jiangxi Provincial Key Laboratory for Ophthalmology, Nanchang, 330006 China

**Keywords:** Problem-based learning, Flipped classroom, Clinical teaching, Ocular trauma

## Abstract

**Background:**

Previous studies have primarily implemented problem-based learning (PBL) or flipped classroom (FC) teaching models in different majors; however, research on the combined PBL-FC teaching method in clinical medicine is scarce. Therefore, we investigated the combined PBL-FC teaching method in teaching ocular trauma on students’ competencies.

**Method:**

About 75 ophthalmology postgraduates were randomly divided into PBL-FC and traditional teaching groups. Students completed pre-and post-class theoretical examinations, skills evaluation, learning ability scales, and feedback questionnaires.

**Results:**

Both groups showed significantly higher theoretical scores and improved learning ability. Feedback questionnaire scores of the PBL-FC group’s postgraduates without clinical experience were significantly higher than the traditional group’s for some items; there was no difference between groups in postgraduates with clinical experience. PBL-FC group’s pre-class preparation time was significantly longer than the traditional group’s, but the post-class review time was significantly shorter. PBL-FC group’s post-class theoretical performance was significantly higher than the traditional group’s. There was no statistical difference between the groups regarding skill operation. Among postgraduates without clinical experience, the PBL-FC group’s skill operation performance was significantly higher than the traditional group’s; for postgraduates with clinical experience, the traditional group’s skill operation performance was significantly higher than the PBL-FC group’s.

**Conclusions:**

PBL-FC teaching is better for students without clinical experience or knowledge of ophthalmic diseases. Meanwhile, traditional teaching is a good choice for students with clinical experience who need more relevant knowledge.

## Introduction

Ophthalmology is an essential and independent subject in medical education and is mainly a morphology-based clinical practice course. The delicate and complex structure of the eye places a high demand on the skills of ophthalmologists. Moreover, owing to the particularity of the ophthalmology course structure, the teaching content provided during traditional theoretical lectures is often abstract and difficult to understand. Therefore, skill training is critical, as it can deepen students' understanding of theoretical knowledge and cultivate their practical abilities. At present, ophthalmology skills training still relies mainly on a traditional lecture-based teaching method, where the lecture is based on the unilateral output of the teacher, and the students' enthusiasm and efficiency are not very high. Previous studies have shown that the average attention span of medical students during lectures is only 10–20 min at the beginning [1]. The most effective way to improve teaching efficiency is to increase the initiative of students in learning, allowing them to interact with the learning materials, participate in the classroom, and cooperate with other students [2, 3]. In recent years, flipped classroom (FC) has become a popular teaching method [4], which is an inverted model of teaching that uses videos, podcasts, or slides to deliver lecture materials outside the classroom [5]. It can improve students’ learning efficiency and deepen their understanding, but teachers may lose the constraints on students [6, 7]. Additionally, the problem-based learning (PBL) method, which is defined as a student-centred pedagogy [8], can also improve students' enthusiasm and efficiency, but it takes a lot of time to find course materials, and difficult problems can cause students to lose their original learning interest and initiative [9, 10].

Some studies have reported the effectiveness and feasibility of PBL combined with FC methods in teaching undergraduate medical students [11, 12], which significantly improve their interest and self-learning ability. Combining the PBL and FC methods to ensure the advantages of the two complement each other can better guide students to think actively and replace the constraints of traditional classrooms in the form of small groups to obtain the best teaching outcome.

However, a combination of PBL and FC methods in ophthalmology education has not yet been explored. In order to improve the quality of ophthalmology education and teaching effect and to stimulate students' independent learning and teamwork ability, we introduced a teaching model combining PBL and FC in ophthalmology skill training and used postgraduates as the research subjects to explore its feasibility and application in ophthalmology education by assessing students’ outcomes.

## Materials and methods

### Participants

We selected 75 professional postgraduates (57 women, 18 men) from the School of Ophthalmology and Optometry, Nanchang University, admitted between September 2018 and 2021. Of the 75 students, 36 were assigned to the PBL-FC group and 39 students to the traditional group. There were 29 first-year postgraduates, 25 s-year postgraduates, and 21 third-year postgraduates. First-year postgraduates were freshmen who had no clinical experience, and the second-and third-year postgraduates were seniors who had clinical experience because of working in rotations in relevant clinical departments. In addition, no participants had previously completed PBL, FC, or PBL combined with FC teaching mode. All students participated in the ophthalmology skill-training programme held by the same lecturer.

### Study design

**Fig. 1:**
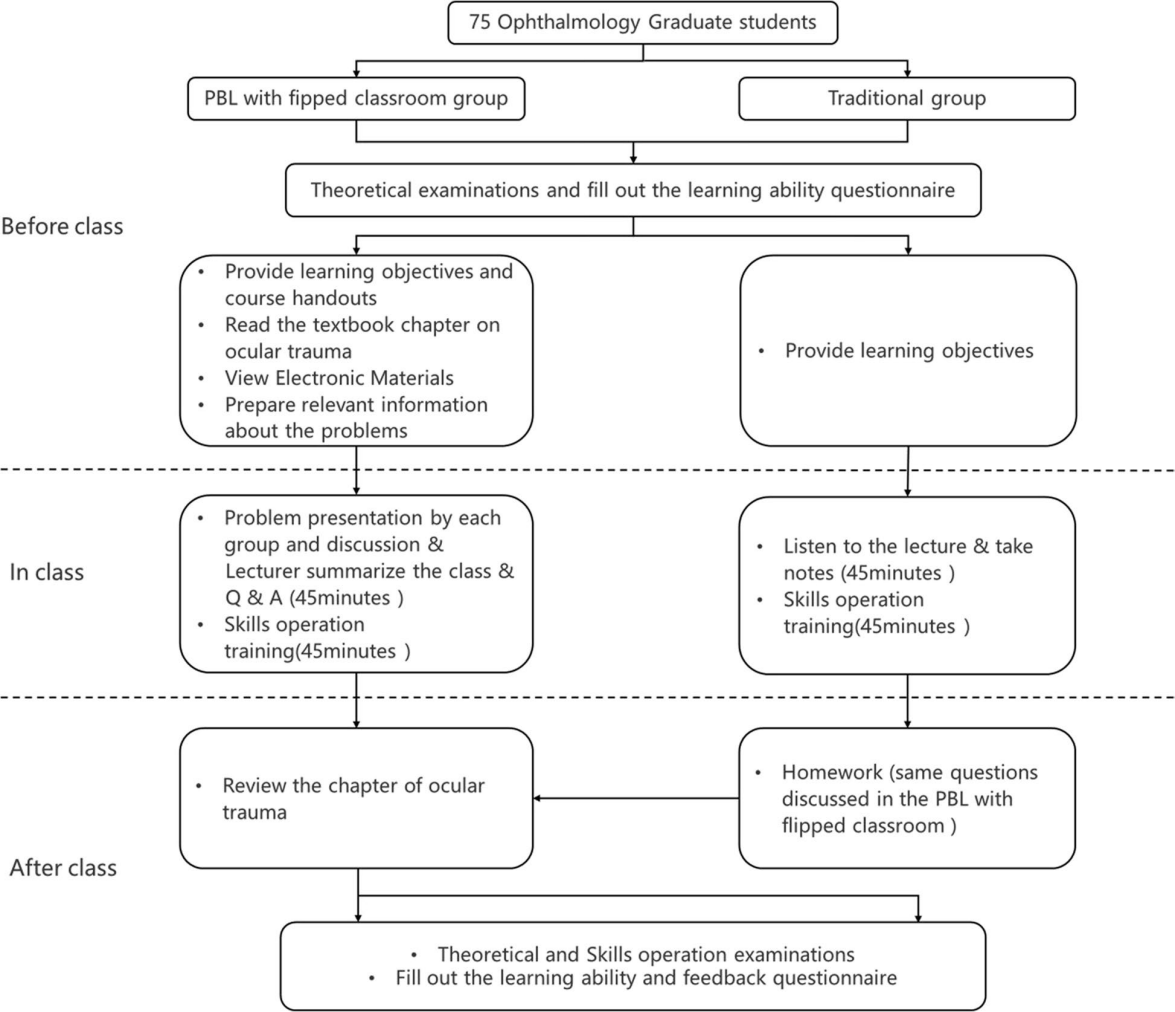
Study flowchart

This was a prospective, randomised, controlled study. The students were randomly divided into two groups. The experimental group adopted a combination of PBL and FC teaching methods, and the control group adopted the traditional teaching method. The textbooks, teaching content, lecturers, and total hours of teaching were the same in both groups. An overview of the flow chart of the study design is presented in Fig. 1.

### Teaching schedule

#### Modules and goals

The complexity of ocular trauma and the need for individualised treatment requires students to have a comprehensive knowledge of ophthalmic diseases and relevant clinical skills. Additionally, the signs and symptoms of ocular trauma are more accessible for students to observe and understand. For this reason, we chose corneal debridement and suturing from the ocular trauma module for this study. This clinical skills course comprises two parts: a theoretical study course and a clinical skills practical course. We referred to the school's teaching outline and requirements and formulated teaching objectives according to the characteristics of the ocular trauma department to help students master corneal debridement and suture.

#### PBL with FC group

##### ***Study group formation***

Divided postgraduates into five groups of six postgraduates each. A communication platform was set up within the group, group leaders were identified, and were given sufficient time to promote interpersonal interactions within the group.

##### ***Pre-class knowledge transfer***

Skill training content and teaching objectives were sent to students before class in the form of text, PPT, e-books, videos, online learning platforms, and other electronic network resources to attract students and enhance their understanding of ophthalmic knowledge and skill training to optimise their learning time.

##### ***Asking questions***

At three days before class, open-ended questions—such as how to treat corneal laceration involving embedded intraocular tissue, considerations for suturing the cornea in different locations, whether corneal laceration suturing must be combined with intraocular injection, how to better refractive suturing, how to treat irregular corneal laceration or corneal tissue loss, different ways of anterior chamber formation, and their advantages and disadvantages—were discussed and independently explored in groups. The aim was to enable students to obtain a fit diagnosis, select an appropriate treatment, and apply what they have learned to analyse and solve clinical problems.

##### ***Training skills in the classroom***

*a) Class discussion*: Students in each group summarised and reported in class, presented their answers, and subsequently discussed clinical questions. Finally, the lecturer summarised and reviewed all the questions from the discussion. *b) Skill training*: After the discussion, students could practice skills through animal models.

#### Traditional group

##### ***Training skills in the classroom***

*a) Classroom theory lectures:* The lecturer explained the precautions of skill operation and relevant theoretical knowledge. *b) Skill training:* After the theoretical class, students drilled the skills operation. They could train skill operations through animal models. If they had questions, they could ask, and the teacher would answer.

##### ***Homework***

The students reviewed the lecture and thought about relevant questions (same as those discussed in the PBL-FC group) and gave written answers within one week. The teacher would provide answers to each question and help students solve the problem if they asked for help.

### Teaching effectiveness evaluation

#### Subjective evaluation

All students self-evaluated their independent learning ability before and after class using the Learning Ability Questionnaire (LAQ) [13]. Students were asked to select the answer that best described their actual situation and were told that the questionnaire had no relationship with their academic performance. Furthermore, all students completed a feedback questionnaire three days after class. The questionnaire was adapted from Ramsden’s curriculum experience questionnaire (CEQ) and Biggs’s research process questionnaire [14]. The questionnaire used a standard five-point Likert scale, with agreement levels of positive items ranging from 5 (strongly agree) to 1 (strongly disagree) and agreement levels of negative items ranging from 1 (strongly agree) to 5 (strongly disagree). Higher scores in both the LAQ and the feedback questionnaire mean better results.

#### Objective evaluation

##### ***Theoretical examinations***

At three days before class, all students conducted a pre-class theoretical examination on ocular trauma, followed by a post-test after three days of the class. All objective evaluations were completed collaboratively by clinicians and skills training lecturers and reviewed by three experts with senior deputy titles or above, with a total score of 100 to assess students' basic understanding of the disease. Additionally, the students were asked to report the time required for pre-class preparation and post-class review.

##### ***Skills evaluation***

Before the skills operation evaluation, we standardised every operation process and key points, assigned values in steps and points, and created the evaluation form. It was tested three days after the class. We used the Delphi method to carry out three rounds of expert questioning, reviewed and modified the evaluation form, and finally completed it with a total score of 100. The same teacher was scored according to a standardised evaluation form.

### Statistical analysis

The data were entered by two persons and verified twice. All statistical analyses were performed using SPSS (version 23.0, Chicago, IL, USA). The measurement data were first tested to determine whether they conform to the normal distribution, using the mean ± standard deviation or the median (interquartile range). If conforming to the normal distribution, the independent sample t-test was used for comparison between groups; otherwise, the Mann–Whitney U test was used. The α was set at 0.05, and *P*-values less than 0.05 were considered statistically significant.

## Results

### Comparison of basic information in two groups

Table 1 compares the basic characteristics of the PBL-FC and traditional groups. There were no significant differences between the two groups in terms of grades, gender, or age. The class attendance rate of the two groups was 100%. All students in the PBL-FC group watched the online lecture video and read the supplementary study materials assigned by the instructor. All students in the traditional group completed and submitted their homework to the instructor on time. The response rate for the questionnaires was 100% in both groups.

**Table 1:** Basic characteristics of two groups

		Clinical experience	PBL-FC group	Traditional group	*P* value
Number			36	39	
Grade	PGY-1	NO	12	17	0.545
	PGY-2	YES	12	13	
	PGY-3		12	9	
Gender	Male		7	11	0.375
	Female		29	28	
Age (in years)			24.17 [23.00–25.00]	23.90[23.00–25.00]	0.353

*PBL-FC* Problem-based learning combined with flipped classroom, *PGY* Postgraduate year

### Comparison of feedback questionnaire between the PBL–FC and traditional groups

We compared feedback questionnaires between the two groups. For postgraduates without clinical experience, there were statistical differences in terms of skills cultivation (*P* = 0.043), appropriate assessment (*P* = 0.004), and independence (*P* = 0.023). The scores of other items in the PBL-FC group were higher than those in the traditional group, but there was no statistical difference between the two groups. For postgraduates with clinical experience, although there was no statistical difference between the two groups, the scores for good teaching design, clear course goals, appropriate assessments, appropriate workload, independence, learning features, summarisation, and total were higher in the traditional group than those in the PBL-FC group (Table 2).

**Table 2:** The comparison of feedback questionnaire of students with and without clinical experience in the PBL-FC group and the traditional group

	Without clinical experience students			With clinical experience students		
	PBL-FC Group	Traditional Group	P	PBL-FC Group	Traditional Group	P
Good teaching design	4.51 ± 0.58	4.41 ± 0.62	0.669	4.46 ± 0.55	4.51 ± 0.46	0.718
Clear course goals	3.53 ± 0.85	3.45 ± 0.78	0.807	3.47 ± 0.79	3.39 ± 0.96	0.759
Skills cultivation	4.65 ± 0.50	4.21 ± 0.60	0.043	4.36 ± 0.50	4.34 ± 0.50	0.901
Appropriate assessment	3.62 ± 0.72	2.61 ± 0.95	0.004	2.79 ± 0.81	3.07 ± 0.99	0.297
Appropriate workload	3.29 ± 0.92	3.07 ± 0.73	0.483	3.05 ± 0.79	3.23 ± 0.80	0.458
Independence	4.63 ± 0.43	4.12 ± 0.70	0.023	4.10 ± 0.51	4.16 ± 0.71	0.764
Learning features	3.90 ± 0.71	3.79 ± 0.63	0.660	3.80 ± 0.52	4.03 ± 0.51	0.149
Summarization	4.52 ± 0.58	4.35 ± 0.67	0.500	3.89 ± 0.52	4.13 ± 0.60	0.157
Total	4.08 ± 0.54	3.75 ± 0.48	0.092	3.75 ± 0.43	3.88 ± 0.47	0.340

*PBL-FC* Problem-based learning combined with flipped classroom

### Comparison of preparation time between the PBL–FC and traditional groups

We compared the pre-and post-class preparation times of the PBL-FC and traditional groups. In general, the pre-class preparation time of the PBL-FC group was significantly longer than that of the traditional group (5.21 ± 3.04 h vs 3.37 ± 2.20 h, *P* = 0.004), while the post-class preparation time of the PBL-FC group was significantly shorter than that of the traditional group (2.03 [1.50–2.50] hours vs 3.15 [2.00–4.00] hours, *P* < 0.001) (Table 3).

**Table 3:** The comparison of the pre- and post-class preparation time, learning ability and test scores of students of PBL-FC group vs. traditional group

		PBL-FC group	Traditional group	*P*-value
Preparation time (in hours)	Pre-class	5.21 ± 3.04	3.37 ± 2.20	0.004
	Post-class	2.03 [1.50–2.50]	3.15 [2.00–4.00]	0.000
Learning ability	Pre-class	4.51 ± 0.43	4.45 ± 0.47	0.595
	Post-class	4.64 [4.39–5.00]	4.56 [4.23–5.00]	0.550
Theoretical performance	Pre-class	66.53 ± 10.61	66.51 ± 9.58	0.665
	Post-class	86.11 ± 7.08	79.10 ± 8.95	0.000
Skills operation performance		82.22 ± 8.16	81.26 ± 11.36	0.676

*PBL-FC* Problem-based learning combined with flipped classroom

**Fig. 2:**
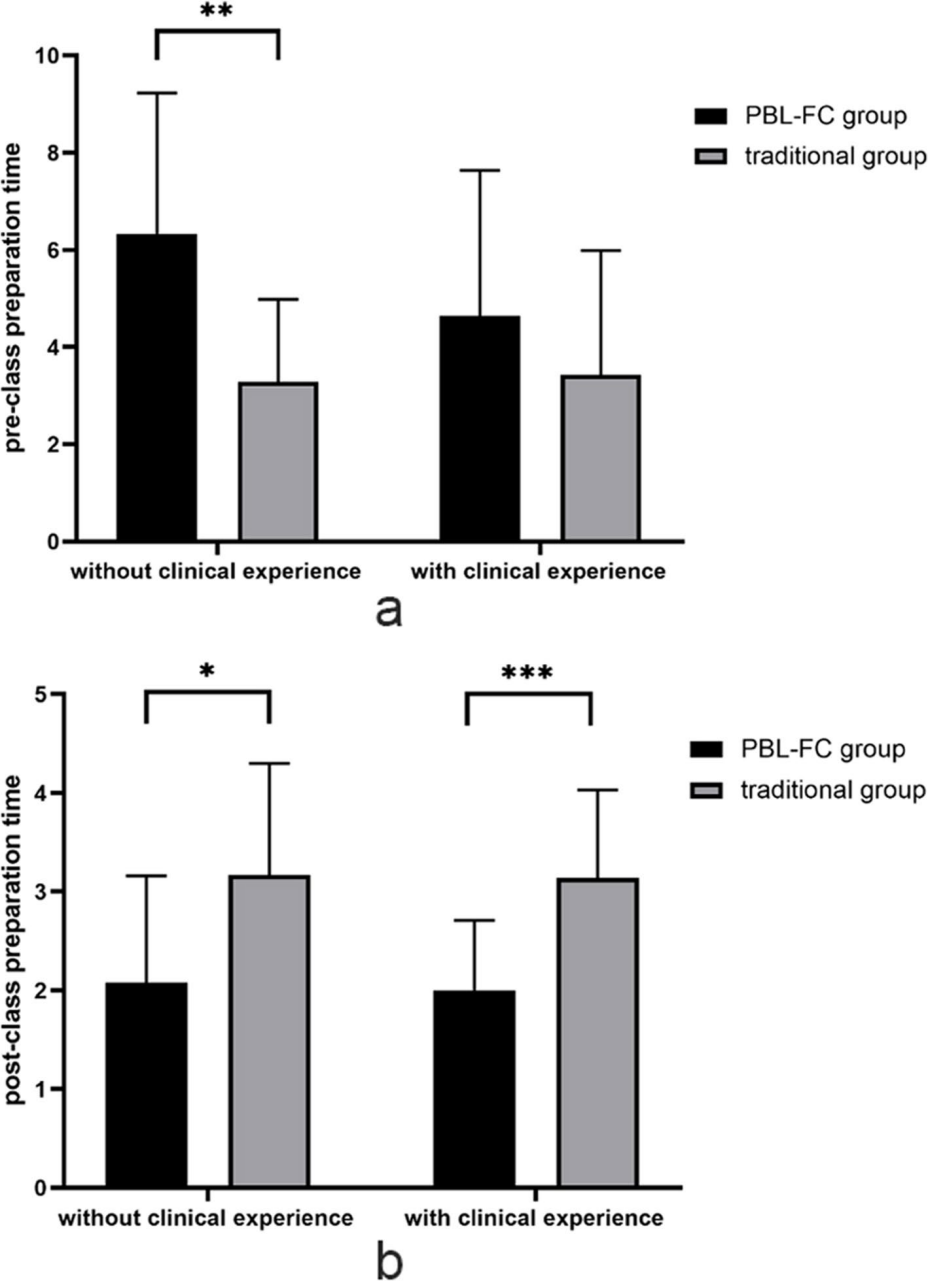
The comparison of preparation time in postgraduates with or without clinical experience in two groups. **a** the comparison of pre-class preparation time in all postgraduates; **b** the comparison of post-class preparation time in all postgraduates. *P < 0.05, **P < 0.01, ***P < 0.001

Additionally, for students without clinical experience, the mean pre-class preparation time was 6.33 ± 2.90 h in the PBL–FC group, whereas for the traditional group, it was 3.29 ± 1.69 h, and there was a statistical difference between the two groups (*p* = 0.001) (Fig. 2a). There was also a statistically significant difference between the two groups in terms of post-class preparation time (*P* = 0.015) (Fig. 2a). For students with clinical experience, there was no statistical difference between the two groups in terms of pre-class preparation time (Fig. 2b). However, the post-class preparation time of the traditional group was significantly longer than that of the PBL-FC group (2.00 ± 0.71 h vs 3.14 ± 0.89 h, *P* < 0.001) (Table 4).

**Table 4:** Comparison of pre-class and after-class evaluation indexes of students with and without clinical experience in the PBL-FC group and the traditional group

		Without clinical experience students			With clinical experience students		
		PBL-FC group	Traditional group	P	PBL-FC group	Traditional group	P
Preparation time (in hours)	Pre-class	6.33 ± 2.90	3.29 ± 1.69	0.001	4.65 ± 3.00	3.43 ± 2.56	0.149
	Post-class	2.08 ± 1.08	3.18 ± 1.13	0.015	2.00 ± 0.71	3.14 ± 0.89	0.000
Learning ability	Pre-class	4.61 ± 0.40	4.49 ± 0.44	0.430	4.46 ± 0.45	4.43 ± 0.50	0.843
	Post-class	4.65 ± 0.45	4.48 ± 0.45	0.327	4.63 ± 0.39	4.61 ± 0.46	0.878
Theoretical performance	Pre-class	64.58 ± 7.82	63.53 ± 4.60	0.681	67.50 ± 11.80	67.05 ± 12.02	0.898
	Post-class	81.25 ± 7.11	73.82 ± 8.01	0.016	88.54 ± 5.80	83.18 ± 7.49	0.009
Skills operation performance		78.75 ± 4.73	72.18 ± 11.36	0.043	83.96 ± 9.01	88.27 ± 4.12	0.042

*PBL-FC* Problem-based learning combined with flipped classroom

### Comparison of learning ability between the PBL-FC and traditional groups

After comparing the PBL-FC and traditional groups’ learning abilities, we found no statistically significant difference between the two groups in terms of pre-and post-class learning abilities (Table 3).

Additionally, for both students with and without clinical experience, though the pre-and post-class learning ability of the PBL-FC group was higher than that of the traditional group, there was no statistical difference between the two groups (Table 4).

### Comparison of theoretical performance between the PBL–FC and traditional groups

There was no statistically significant difference between the two groups in terms of pre-class theoretical performance. The post-class theoretical performance of the PBL-FC group was significantly higher than that of the traditional group (86.11 ± 7.08 vs 79.10 ± 8.95, *P* < 0.001). All the data are shown in Table 3.

**Fig. 3:**
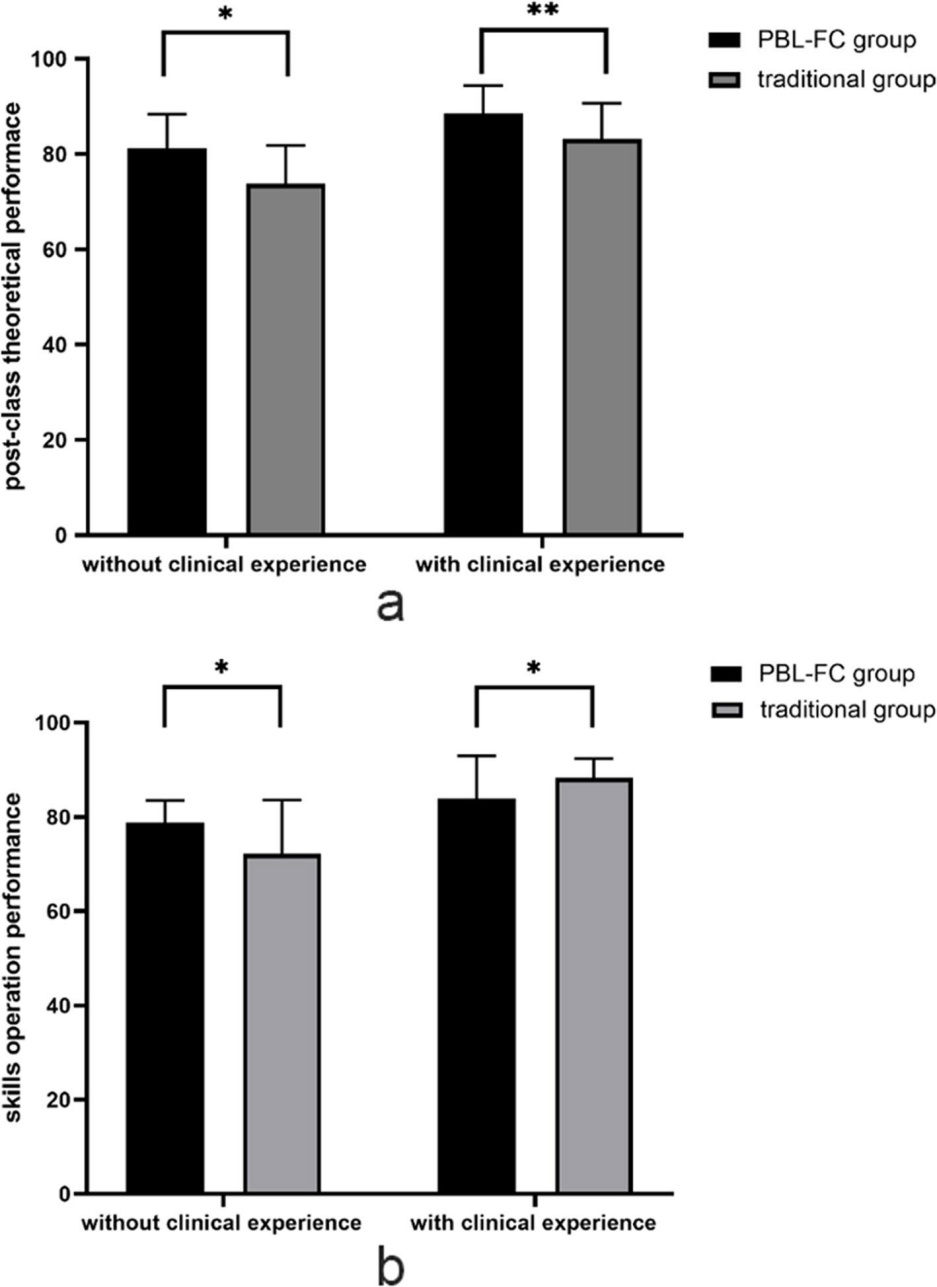
Comparison of post-class theoretical performance and skills operation performance in two groups. **a** the comparison of post-class theoretical scores in all the postgraduates; **b** the comparison of skills operation scores in all the postgraduates. *P < 0.05, **P < 0.01

Additionally, for both students with and without clinical experience, there was no statistical difference between the two groups in terms of pre-class theoretical performance. However, the PBL-FC group had better theoretical performance than the traditional group for students with no clinical experience (*p* = 0.016) as well as for students with clinical experience (*p* = 0.009) (Table 4) (Fig. 3a).

### Comparison of skills operation between the PBL–FC and traditional groups

A comparison of the PBL-FC and traditional groups’ skill operation scores revealed that, in general, the skill operation performance in the PBL-FC group was higher than that in the traditional group, with no statistical difference (*P* = 0.676). The results are shown in Table 3. However, we found that students without clinical experience had better skill operation performance after the PBL-FC teaching method compared to the traditional teaching method (78.75 ± 4.73 vs 72.18 ± 11.36, *P* = 0.043), while students with clinical experience had the opposite result, meaning the skill operation performance of the traditional group was better than the PBL-FC group for students with clinical experience (83.96 ± 9.01 vs 88.27 ± 4.12, *P* = 0.042) (Table 4) (Fig. 3b).

### Comparison of pre-and post-class theoretical performance and learning ability

There was no statistical difference in learning abilities between students with and without clinical experience for the PBL-FC and traditional groups. However, theoretical performance in students with and without clinical experience was significantly different from pre- to post-class in the two groups (*p* < 0.001). All the data are presented in Table 5.

**Table 5:** The comparison of the pre- and post-class test scores in two groups

		Pre-class	Post-class	*P*-value
Learning ability	PBL-FC group	4.51 ± 0.43	4.64 ± 0.40	0.057
	Traditional group	4.45 ± 0.47	4.56 ± 0.45	0.218
Theoretical performance	PBL-FC group	66.53 ± 10.61	86.11 ± 7.08	0.000
	Traditional group	66.51 ± 9.58	79.10 ± 8.95	0.000

*PBL-FC* Problem-based learning combined with flipped classroom

## Discussion

Ocular trauma cases often cover a wide range of ocular injuries, which require students to have a comprehensive knowledge of ophthalmic diseases, including fundus disease, glaucoma, cataract, iridodonesis, vitreous haemorrhage, and retinal detachment [15]. Since the clinical signs and symptoms of ocular trauma are more accessible for students to observe and understand, ocular trauma is a suitable topic for the implementation of the FC approach compared to other topics. Consistently, studies have shown that students evaluate the ocular trauma-FC more positively than the glaucoma-FC [16]. Additionally, PBL has established a small-group learning mode, which features more thorough teacher–student communication and, thus, can achieve personalised teaching goals (Refai D, Thompson JL: The significance of problem-based learning in the development ofenterprise skills for pharmacy students in UK HEI, unpublished) [17]. Given the advantages of both approaches and the combined considerations, we chose the ocular trauma module to apply PBL combined with the FC teaching approach for this study.

All students and teachers completed the questionnaires at the end of the course, with a response rate of 100%. Based on the questionnaire feedback, we found that the PBL-FC teaching model was highly welcomed by participating first-year postgraduates. It may be because: (1) First-year postgraduates have no in-depth knowledge of clinical ophthalmology, and PBL-FC teaching is a stimulating teaching method. Students’ interest in learning is stimulated by an actual clinical case and the relevant skill’s operation assigned. This increased interest in both emotional and cognitive levels is beneficial for learning [18, 19]. (2) The second-year and third-year postgraduates of study have little free time due to heavy clinical tasks, and the preparation time before PBL-FC classes is relatively long. As they already have a lot of knowledge about ophthalmology, the stimulating and interesting aspects of PBL-FC teaching are no different from traditional teaching for them.

No statistical differences were found between the PBL-FC and traditional groups in the pre-class test, implying that no differences were observed in the basic knowledge of the ocular trauma module between the two groups, which is consistent with the results of Lin [1]. However, in our study, we found significantly longer pre-class preparation times for the PBL-FC group than for the traditional teaching group among first-year postgraduates without clinical experience but not among second-and third-year postgraduates with clinical experience. This could be due to the lack of clinical experience and unfamiliarity with postgraduate studies, which probably increased the pre-class preparation time. Although the pre-class preparation time was increased for first-year postgraduates without much clinical experience, the feedback questionnaire reflects that these students do not feel that the preparation work was too heavy. Additionally, for postgraduates in the PBL-FC group, their post-class preparation time was significantly shorter than that of the traditional group. Compared to the traditional group, the postgraduates in the PBL-FC group did not complain that the workload was heavy.

Furthermore, students were provided with a post-class test that assessed their acquisition of ocular trauma knowledge and their ability to apply what they had learned, including theoretical knowledge and a clinical skills test. Although the post-class test showed that the theoretical scores of the PBL-FC group were significantly higher than those of the traditional group, both groups showed significant improvement in their post-theoretical scores compared to the pre-theoretical scores. The above results show that while both approaches are helpful for students’ theoretical acquisition, the FBL-PC teaching method is more effective. Hu et al. [20] also found a significant improvement in examination scores associated with the PBL-FC teaching mode. However, there were some differences in the skill operation performance. We considered that in the first-year postgraduates without clinical experience, detailed PBL-FC teaching could give the students more time to review the information and watch the related skills operation videos before the class to make up for the lack of clinical experience and unfamiliarity with ophthalmology knowledge. Thus, in the first-year postgraduates, the skill operation scores of the PBL-FC group were significantly higher than those of the traditional group. Chiu et al. [21] also found that students without clinical experience in the FC group had a better improvement in skills operation. In contrast, for second-and third-year postgraduates with clinical experience who had more clinical knowledge and experience already had a certain understanding of the relevant clinical skills operation during preparation to review the information before class. All the learning processes ranged from easy to difficult. Since the second-and third-year students had already gained basic clinical ophthalmology knowledge, the effect of skill improvement was not very significant, even through the PBL-FC method. Instead, they may require more of the clinical experience and esoteric knowledge taught by teachers in the traditional class. This is the reason why the skill operation performance of the traditional group was higher in the second-and third-year postgraduates.

Previous studies have shown attempts to implement either PBL or FC teaching models in various colleges and university majors [8, 16, 22]; however, little attention has been paid to the combined PBL-FC teaching model in clinical medicine. This study fills the gap in literature by evaluating the combined teaching model and applying it to ophthalmology. Furthermore, it focuses on theoretical knowledge improvement and the inclusion of clinical operation skills learning, along with subgroup analysis of different types of students, including first-, second-, and third-year postgraduates.

In conclusion, this study confirmed that the combined PBL-FC teaching method could improve overall understanding and absorption of professional knowledge for medical postgraduates and promote teacher-student interaction and teamwork ability. Simultaneously, it is beneficial to improve the clinical operation skills of inexperienced first-year postgraduates. The combined PBL-FC teaching method is better for students who have no clinical experience and are unfamiliar with diseases and related skills operations. However, the traditional teaching method is a better choice for students who have some clinical experience and require more relevant experience taught by teachers.

Nevertheless, there are some limitations to this study. First, although we achieved positive results in the implementation of PBL-FC teaching for 75 postgraduates, the sample size was relatively small. Thus, the next step for this research will be to extend it to the clinical teaching of a larger group of postgraduates majoring in ophthalmology. Second, the number of classes was small, which may have caused an inaccurate assessment of the combined PBL-FC teaching model. For future research, we plan to conduct multiple PBL-FC teaching on multiple teaching topics and re-evaluate them in a broader range of ophthalmology postgraduate students.

## Data Availability

The datasets used and/or analysed during the current study are available from the corresponding author on reasonable request.

## References

[1] Lin Y, Zhu Y, Chen C, Wang W, Chen T, Li T, Li Y, Liu B, Lian Y, Lu L (2017). Facing the challenges in ophthalmology clerkship teaching: is flipped classroom the answer?. PLoS One.

[2] Freeman S, Eddy SL, McDonough M, Smith MK, Okoroafor N, Jordt H, Wenderoth MP (2014). Active learning increases student performance in science, engineering, and mathematics. Proc Natl Acad Sci USA.

[3] Prober CG, Heath C (2012). Lecture halls without lectures–a proposal for medical education. N Engl J Med.

[4] Lauermann JL, Treder M, Merté RL, Schloßbauer A, Becker JC, Marschall B, Eter N, Brücher VC (2021). "Flipped classroom"-A future concept for student teaching in ophthalmology?. Der Ophthalmologe.

[5] Strayer JF (2012). How learning in an inverted classroom influences cooperation, innovation and task orientation. Learn Environ Res.

[6] Khanova J, Roth MT, Rodgers JE, McLaughlin JE (2015). Student experiences across multiple flipped courses in a single curriculum. Med Educ.

[7] Foldnes N (2016). The flipped classroom and cooperative learning: Evidence from a randomised experiment. Act Learn High Educ.

[8] Smits PB, de Buisonjé CD, Verbeek JH, van Dijk FJ, Metz JC, ten Cate OJ (2003). Problem-based learning versus lecture-based learning in postgraduate medical education. Scand J Work Environ Health.

[9] Amalba A, Abantanga FA, Scherpbier A, van Mook W (2019). Trainees' preferences regarding choice of place of work after completing medical training in traditional or problem-based learning/community-based education and service curricula: a study in Ghanaian medical schools. Rural Remote Health.

[10] Jones RW (2006). Problem-based learning: description, advantages, disadvantages, scenarios and facilitation. Anaesth Intensive Care.

[11] Chen Q, Yue W (2020). Application of flipped classroom and problem-based learning in education of precision medicine. Reasearch Exploration Lab.

[12] Lei Y, Lv P, Zhao J, Li Q (2017). Application of task-driven flipped classroom in genetics experiment course. Basic Med Educ.

[13] Zhu L, Lian Z, Engström M (2020). Use of a flipped classroom in ophthalmology courses for nursing, dental and medical students: a quasi-experimental study using a mixed-methods approach. Nurse Educ Today.

[14] Ramsden P (1991). A performance indicator of teaching quality in higher education: The Course Experience Questionnaire. Stud High Educ.

[15] Shukla B, Agrawal R, Shukla D, Seen S (2017). Systematic analysis of ocular trauma by a new proposed ocular trauma classification. Indian J Ophthalmol.

[16] Tang F, Chen C, Zhu Y, Zuo C, Zhong Y, Wang N, Zhou L, Zou Y, Liang D (2017). Comparison between flipped classroom and lecture-based classroom in ophthalmology clerkship. Med Educ Online.

[17] Koh GC, Khoo HE, Wong ML, Koh D (2008). The effects of problem-based learning during medical school on physician competency: a systematic review. CMAJ.

[18] Harp S, Mayer R (1997). The role of interest in learning from scientific text and illustrations: on the distinction between emotional interest and cognitive interest. J Educ Psychol.

[19] Nandi PL, Chan JN, Chan CP, Chan P, Chan LP (2000). Undergraduate medical education: comparison of problem-based learning and conventional teaching. Hong Kong Med J.

[20] Hu X, Zhang H, Song Y, Wu C, Yang Q, Shi Z, Zhang X, Chen W (2019). Implementation of flipped classroom combined with problem-based learning: an approach to promote learning about hyperthyroidism in the endocrinology internship. BMC Med Educ.

[21] Chiu HY, Kang YN, Wang WL, Huang HC, Wu CC, Hsu W, Tong YS, Wei PL (2018). The effectiveness of a simulation-based flipped classroom in the acquisition of laparoscopic suturing skills in medical students-a pilot study. J Surg Educ.

[22] Khaki AA, Tubbs RS, Zarrintan S, Khamnei HJ, Shoja MM, Sadeghi H, Ahmadi M (2007). The first year medical students' perception of and satisfaction from problem-based learning compared to traditional teaching in gross anatomy: introducing problem-based anatomy into a traditional curriculum in Iran. Int J Health Sci.

